# A Pruning-Based Disk Scheduling Algorithm for Heterogeneous I/O Workloads

**DOI:** 10.1155/2014/940850

**Published:** 2014-03-23

**Authors:** Taeseok Kim, Hyokyung Bahn, Youjip Won

**Affiliations:** ^1^Department of Computer Engineering, Kwangwoon University, Seoul 139-701, Republic of Korea; ^2^Department of Computer Science and Engineering, Ewha University, Seoul 120-750, Republic of Korea; ^3^Division of Electrical and Computer Engineering, Hanyang University, Seoul 133-791, Republic of Korea

## Abstract

In heterogeneous I/O workload environments, disk scheduling algorithms should support different QoS (Quality-of-Service) for each I/O request. For example, the algorithm should meet the deadlines of real-time requests and at the same time provide reasonable response time for best-effort requests. This paper presents a novel disk scheduling algorithm called G-SCAN (Grouping-SCAN) for handling heterogeneous I/O workloads. To find a schedule that satisfies the deadline constraints and seek time minimization simultaneously, G-SCAN maintains a series of candidate schedules and expands the schedules whenever a new request arrives. Maintaining these candidate schedules requires excessive spatial and temporal overhead, but G-SCAN reduces the overhead to a manageable level via pruning the state space using two heuristics. One is grouping that clusters adjacent best-effort requests into a single scheduling unit and the other is the branch-and-bound strategy that cuts off inefficient or impractical schedules. Experiments with various synthetic and real-world I/O workloads show that G-SCAN outperforms existing disk scheduling algorithms significantly in terms of the average response time, throughput, and QoS-guarantees for heterogeneous I/O workloads. We also show that the overhead of G-SCAN is reasonable for on-line execution.

## 1. Introduction

As an increasingly large variety of applications are developed and equipped in modern computer systems, there is a need to support heterogeneous performance requirements for each application simultaneously. For example, a deadline-guaranteed service is required for real-time applications (e.g., audio or video playback), while reasonable response time and high throughput are important for interactive best-effort applications (e.g., web navigation or file editing). Since these applications require different QoS- (Quality-of-Service-) guarantees, an efficient disk scheduling algorithm that can deal with heterogeneous I/O requests is needed.

Due to the mechanical overhead for accessing data in hard disk-based storage systems, I/O scheduling has been a long-standing problem for operating system and storage system designers. An optimal I/O schedule in the traditional disk scheduling domain usually refers to a sequence of requests that has minimum scanning time. In order to find this optimal schedule, all possible request sequences need to be searched. This is a complicated searching problem which is known as NP hard [1]. The location of each requested block is represented as cylinder, head, and sector information. The distance between two points in this three-dimensional space does not satisfy the Euclidean property. Therefore, to obtain an optimal solution, we should enumerate all possible orderings of a given set of I/O requests. For example, if there are *n* requests in the I/O request queue, the number of all possible combinations is *n factorial*. Unfortunately, finding an optimal schedule from this huge searching space is not feasible due to the excessive spatial and temporal overhead. For this reason, most practical scheduling algorithms simply use deterministic heuristic approaches instead of searching huge spaces.

Unlike traditional scheduling problems, scheduling in heterogeneous workload environments is even more complicated because it should meet the deadlines of real-time requests and provide reasonable response times for best-effort requests, simultaneously. This implies the necessity of scanning huge search spaces rather than simple deterministic processes as in traditional scheduling problems. Y.-F. Huang and J.-M. Huang presented a new approach called MS-EDF (Minimizing Seek time Earliest Deadline First) that effectively reduces the huge state space to a feasible extent through the branch-and-bound strategy [2]. Though MS-EDF shows superior performances, it has some limitations. First, MS-EDF handles requests in a batch manner and thus it cannot be practically used for on-line scheduling. Second, MS-EDF considers only real-time requests, so adopting it directly to the domain of heterogeneous workload environments is not possible.

In this paper, we present a novel disk scheduling algorithm called G-SCAN (Grouping-SCAN) for handling heterogeneous workloads. G-SCAN resolves the aforementioned problems by employing an on-line mechanism and several rules exploiting the QoS requirements of I/O requests. Specifically, G-SCAN first arranges requests in the queue by the SCAN order and then clusters adjacent best-effort requests into a group to schedule them together. Then, G-SCAN reduces the huge searching space to a reasonable extent by pruning unnecessary schedules using the branch-and-bound strategy. Experimental results show that G-SCAN performs better than existing disk scheduling algorithms in terms of average response time, throughput, and QoS-guarantees for heterogeneous workload environments. We also show that the space and time overhead of G-SCAN is reasonable for on-line execution.

The remainder of this paper is organized as follows.  Section 2 presents the state of the art of disk scheduling algorithms. In Section 3, the proposed scheduling algorithm, namely, G-SCAN, is explained in detail. The validation of G-SCAN is described in Section 4 by extensive experiments. Finally, we conclude this paper in Section 5.

## 2. Related Works

Since disk-based storage is always one of the performance bottlenecks in computer systems, disk scheduling algorithms have been studied extensively in the last few decades. Recently, as disks are used as the storage for multimedia data with soft real-time constraints, I/O scheduling problems have become more complicated. In this section, we classify existing disk scheduling algorithms into several classes according to the design purpose.

The first class is throughput-oriented scheduling algorithms. This class of algorithms concentrates on the optimization of disk head movement. SSTF [3], SATF [4], SCAN [5], and C-SCAN [5] are such examples. Of these, SSTF and SATF require an elaborate disk model in order to predict disk seek time or access time, which are not required for SCAN-like algorithms. This is the reason why SCAN and its variants such as C-SCAN are widely used in commodity operating systems. Note that this class of algorithms does not consider the priority of requests, and thus they do not have the function of real-time supports.

The second class is real-time scheduling algorithms, and they again can be classified into two categories: deadline-based algorithms and round-based algorithms. Deadline-based algorithms aim at servicing I/O requests within given deadlines. EDF (Earliest Deadline First) is a representative algorithm in this category [6]. The concept of EDF comes from the real-time CPU scheduling technique. As EDF focuses only on deadlines, it exhibits poor performance in terms of disk head movement. Hence, a number of policies have been proposed to reduce the disk head movement of EDF. They include SCAN-EDF [7, 8], SSEDO/SSEDV [9], FD-SCAN [10], SCAN-RT [11], DM-SCAN [12], and Kamel's algorithm [13]. Most of these algorithms combine the features of EDF and SCAN in order to meet the deadlines of real-time requests and maximize the disk utilization. However, since this approach is based on priority, they may induce the starvation of requests with low priorities.

Round-based algorithms are designed for continuous media data and they exploit the periodicity of data retrieval in audio/video playback. They first define the size of round and service all I/O requests before the round expires. Rangan's algorithm [14], Grouped Sweep Scheduling (GSS) [15], Preseeking Sweep algorithm [16], and Chen's algorithm [17] can be classified into this category. These algorithms primarily focus on the efficiency of underlying resources rather than explicitly consider the deadlines of real-time requests. Instead, deadlines could be satisfied in the round-based algorithms by careful load control through the admission control mechanism. These algorithms mandate the in-depth knowledge of disk internals, such as the number of cylinders, the number of sectors per cylinder, and the curve function of seek distance and seek time, which are not usually accessible from the operating system's standpoint.

The third class is algorithms for heterogeneous I/O workloads. During the last years, handling heterogeneous workloads in a single storage device has become an important issue as integrated file systems get momentum as the choice for next generation file systems. The most famous work is Cello [18]. Shenoy and Vin proposed the Cello disk scheduling framework using two-level disk scheduling architectures: a class-independent scheduler and a set of class-specific schedulers. Cello first classifies disk requests into several classes based on their requirement of service. Then it assigns weights to the application classes and allocates disk bandwidth to the application classes in proportion to their weights. Won and Ryu [19], Wijayaratne and Narasimha Reddy [20], and Tsai et al. [21] also proposed scheduling strategies for heterogeneous workloads.

More recently, general frameworks that can control different scheduling parameters such as deadline, priority, and disk utilization were presented. For example, Mokbel et al. proposed Cascaded-SFC which provides a unified framework that can scale scheduling parameters [22]. It models multimedia I/O requests as points in multidimensional subspaces, where each dimension represents one of the parameters. These general scheduling frameworks require many tuning parameters to be set by the system itself or end users. Povzner et al. proposed Fahrrad that allows applications to reserve a fixed fraction of a disk's utilization [23]. Fahrrad reserves disk resources in terms of the utilization by using disk time utilization and period. They also proposed a multilayered approach called Horizon to manage QoS in distributed storage systems [24]. Horizon has an upper-level control mechanism to assign deadlines to requests based on workload performance targets and a low-level disk I/O scheduler deigned to meet deadlines while maximizing throughput.

Most of the aforementioned scheduling algorithms employ deterministic approaches. “Deterministic” here means that the algorithms maintain only a single schedule to be actually executed, and each time a new request arrives the schedule is simply updated. Though deterministic algorithms are effective for fast on-line processing, they have difficulty in maximizing the performance. For example, a new request in the future may change the order of the optimal schedule of existing requests, but this cannot be reflected in deterministic algorithms. Y.-F. Huang and J.-M. Huang presented MS-EDF (Minimizing Seek time Earliest Deadline First) for multimedia server environments that is not a deterministic algorithm [2]. They recognized I/O scheduling as an NP-hard problem and made an initial attempt to reduce the searching space. However, MS-EDF is a kind of off-line algorithm, so it cannot be adopted directly as the on-line scheduler of heterogeneous workload environments.  Table 1 lists a summary of various disk scheduling algorithms.

## 3. G-SCAN: A Pruning-Based Disk Scheduling

### 3.1. Goal and Assumptions

Our goal is to design a disk scheduling algorithm that satisfies the deadline requirement of real-time requests and at the same time minimizes the seek distance of the disk head as much as possible. In addition to this, the scheduling algorithm should be feasible to be implemented; that is, the execution overhead of the algorithm should be reasonable in terms of both space and time for on-line execution.

We first classify I/O requests into two classes: real-time requests and best-effort requests. We assume that each I/O request *R*_*i*_ consists of (*d*_*i*_, *t*_*i*_), where *d*_*i*_ is the deadline and *t*_*i*_ is the track number of *R*_*i*_ on the disk. Real-time requests have their own deadlines and they can be periodic or aperiodic. Best-effort requests have no specific deadlines, and thus we assume their deadlines to be infinite. We also assume that all requests are independent, which implies that a request does not synchronize or communicate with other requests and all requests are nonpreemptive while being serviced in the disk.

Since G-SCAN is an on-line scheduling mechanism, it should decide the schedule of requests immediately when a new request arrives or the service of a request is completed. Though G-SCAN expands existing schedules whenever an arrival or a departure of a request occurs, it reduces the searching space significantly by grouping and branch-and-bound strategies.

### 3.2. Grouping of Best-Effort Requests

We group adjacent best-effort requests and consider them as a single request to service them together. To do this, we arrange the requests in the queue by the SCAN order and then cluster adjacent best-effort requests into a group. Since best-effort requests have no deadlines, it is reasonable to service them together within a group. This grouping reduces the huge searching space significantly by removing unnecessary combinations.

**Figure 1:**
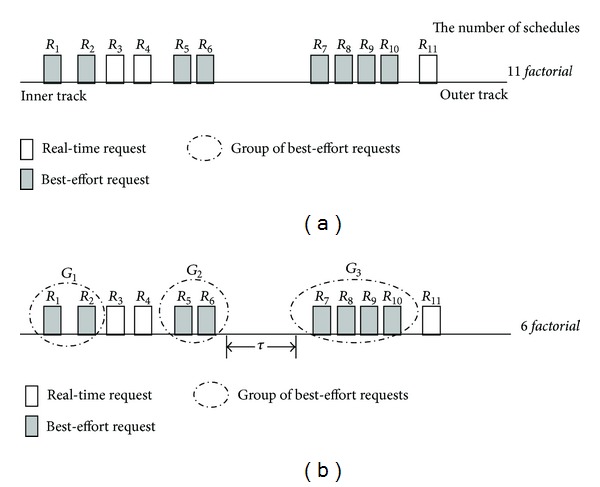
An example of grouping best-effort requests located closely to each other. In (a), the number of all possible combinations before grouping is 11* factorial*. On the other hand, as in (b), the number of all possible combinations after grouping becomes 6* factorial*.

Figure 1 illustrates the grouping of adjacent best-effort requests. There are 11 requests sorted by the SCAN order, and the searching space is 11* factorial *as shown in Figure 1(a). In this example, for best-effort requests *R*_7_, *R*_8_, *R*_9_, and *R*_10_, the ordered schedule *R*_7_ → *R*_8_ → *R*_9_ → *R*_10_ or *R*_10_ → *R*_9_ → *R*_8_ → *R*_7_ is always superior to the nonordered schedules such as *R*_7_ → *R*_10_ → *R*_9_ → *R*_8_ in terms of the seek distance.

Figure 1(b) shows the state after grouping adjacent best-effort requests. Basically, G-SCAN clusters all best-effort requests between two real-time requests into a single group. However, if the seek distance between any two best-effort requests is too long, they are not put together into the same group. This is because a group that spans too long distance may decrease the possibility of finding good schedules. Hence, we put any two adjacent best-effort requests whose distance is below the threshold *τ* into the same group, where *τ* is an experimental parameter. In Figure 1(b), *R*_6_ and *R*_7_ belong to separate groups because their distance is longer than *τ*. If *τ* is large, the number of possible schedules decreases and thus the searching space becomes smaller, but the possibility of finding the best schedule also decreases.

When a new request arrives at the queue, G-SCAN groups it by the aforementioned method. If the new request is a best-effort one, it may be merged into an existing group, bridge a gap between two groups, or create a new group. On the other hand, if the new request is a real-time one, it may split an existing group or just be inserted by the SCAN order without any specific actions.

### 3.3. The Branch-and-Bound Strategy

To reduce the searching space even more, we employ the branch-and-bound strategy similar to the approach of Y.-F. Huang and J.-M. Huang [2]. The branch-and-bound strategy is an algorithmic technique to find an optimal solution in combinatorial optimization problems by keeping the best solution found so far. If a partial solution cannot improve at best, it is pruned not to produce unnecessary combinations any more. Since I/O scheduling is a typical combinatorial optimization problem, the branch-and-bound strategy can be effectively used for this problem.

**Figure 2:**
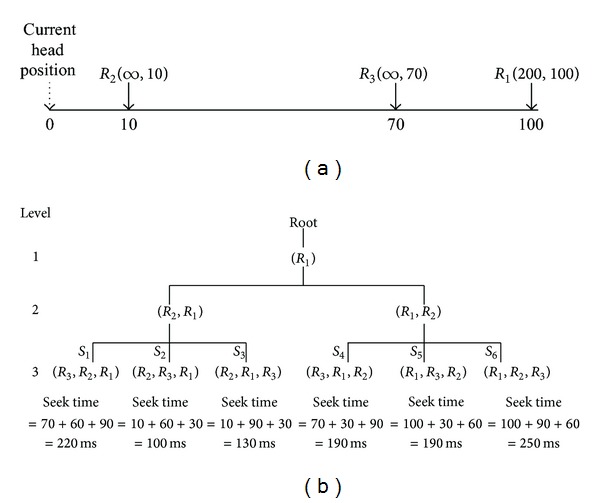
An example for pruning. There are three requests *R*_1_, *R*_2_, and *R*_3_ in the queue. Schedules *S*_1_ and *S*_6_ can be pruned because *S*_1_ misses the deadline 200 ms of *R*_1_, and *S*_6_ incurs too long seek times.

We cut down two kinds of unnecessary schedules from huge searching spaces using the QoS requirements of heterogeneous workloads. The first class is schedules that have any deadline missed request and the second class is schedules that incur too long seek time.  Figure 2 illustrates an example of the cutting-down process. Let us assume that *R*_1_ is a real-time request with the deadline of 200 ms, and *R*_2_ and *R*_3_ are best-effort requests. In this example, for simplicity, we assume that the seek time of track-to-track is 1 ms and the seek time is proportional to the track distance of the requests. We also assume that the rotational latency for each request is constant and do not consider the transfer time because it is very small compared to the seek time and the rotational latency. Note that these factors are considered in the experiment section.

In Figure 2(b),* level* denotes the number of requests in the queue. For example, when the level is 3, the searching space is 3* factorial*. Among all possible combinations, some schedules can be removed from this tree structure. For example, schedule *S*_1_ can be removed because request *R*_1_ in schedule *S*_1_ cannot meet its deadline of 200 ms. Note that any schedules inherited from this schedule cannot also satisfy the deadline constraints, which we will show in Theorem 1. Schedule *S*_6_ can also be removed because it incurs too long seek time. A concrete yardstick for “too large” here will be given more clearly in Theorem 2. As a result, practical searches for finding the best schedule can be performed only with the remaining schedules. An optimal schedule in this example is *S*_2_, because its seek time is shortest among the schedules satisfying the deadline requirement of real-time requests.

Now, we will show why the two classes of schedules and their successors cannot produce an optimal schedule and thus can be pruned. These two pruning conditions can be proved through the following two theorems.

**Theorem 1**

If a schedule does not meet the deadline of any real-time request, then all new schedules inherited from that schedule will not also meet the deadlines.

**Proof**

Let us assume that there is a schedule with the request order (…, *R*_*i*_, …), where 1 ≤ *i* ≤ *n*, that cannot meet the deadline of *R*_*i*_. When a new request *R*_*n*+1_ arrives, G-SCAN expands existing schedules by inserting *R*_*n*+1_ into positions either before or after *R*_*i*_, that is, (…, *R*_*n*+1_, …, *R*_*i*_, …) or (…, *R*_*i*_, …, *R*_*n*+1_, …). In the latter case that *R*_*n*+1_ is serviced later than *R*_*i*_, the service time of *R*_*i*_ does not change at all, and thus *R*_*i*_ still misses the deadline. In the former case that *R*_*n*+1_ is serviced earlier than *R*_*i*_, the seek time of *R*_*i*_ will not obviously be reduced. Hence, the schedule cannot meet the deadline of *R*_*i*_.

**Theorem 2**

Assume that there are *n* requests in the queue and the seek time of a schedule *S*_*i*_(*n*) is longer than that of an optimal schedule *S*_opt_(*n*) for a full sweep time of the disk head. Then, any schedule *S*_*i*_(*n* + 1) expanded from *S*_*i*_(*n*) due to the arrival of a new request cannot be an optimal schedule.

**Proof**

Let *C*_opt_(*n*) and *C*_*i*_(*n*) be the seek time of *S*_opt_(*n*) and *S*_*i*_(*n*), respectively. Then, by the assumption of this theorem, the following expression holds:

(1) $$\begin{array}{c} C_{i}\left(n\right)-\;C_{\text{opt}}\left(n\right)>C_{\text{sweep}}, \end{array}$$

where *C*_sweep_ is the seek time of a full disk head sweep. Similarly, let *S*_opt_(*n* + 1) be an optimal schedule after arriving (*n* + 1)th request, and let *C*_*i*_(*n* + 1) and *C*_opt_(*n* + 1) be the seek time of *S*_*i*_(*n* + 1) and *S*_opt_(*n* + 1), respectively. Since *S*_*i*_(*n* + 1) is inherited from *S*_*i*_(*n*) by including a new request, the following expression holds:

(2) $$\begin{array}{c} C_{i}\left(n+1\right)\geq C_{i}\left(n\right). \end{array}$$

Also, expression (3) is satisfied because an additional seek time for the new request is not longer than the seek time of a full disk head sweep in the case of the optimal algorithm:

(3) $$\begin{array}{c} C_{\text{opt}}\left(n\right)+C_{\text{sweep}}\geq C_{\text{opt}}\left(n+1\right). \end{array}$$

Through expressions (1), (2), and (3), the following expression is derived:

(4) $$\begin{array}{c} C_{i}\left(n+1\right)\;>C_{\text{opt}}\left(n+1\right). \end{array}$$

This implies that any schedule *S*_*i*_(*n* + 1) inherited from *S*_*i*_(*n*) which satisfies expression (1) cannot have shorter seek time than that of *S*_opt_(*n* + 1). Hence, *S*_*i*_(*n* + 1) cannot be an optimal schedule.

The above two pruning conditions are devised to reduce the searching space when a new request arrives at the queue. Similarly, it is also possible to reduce the searching space when a request is removed from the queue. Specifically, when the disk becomes ready to perform a new I/O operation, G-SCAN selects the best schedule among the candidate schedules and dispatches the first request in that schedule. This makes schedules not beginning with the selected request meaningless and thus they can be pruned. Details of this pruning condition are explained in Theorem 3.

**Theorem 3**

When a request *R*_*i*_ leaves from the queue to be serviced, any schedules that do not begin with *R*_*i*_ can be pruned.

**Proof**

Let us suppose that an optimal schedule with *n* requests is *S*_opt_(*n*) and the first request in *S*_opt_(*n*) is *R*_*i*_. When the disk becomes ready to service a request, the scheduling algorithm selects *S*_opt_(*n*) and removes *R*_*i*_ from the queue to service it. In this case, all schedules that do not begin with *R*_*i*_ can be removed from the searching space because schedules inherited from them as well as themselves are all invalid. On the other hand, schedules beginning with *R*_*i*_ are not pruned but remain in the tree structure though they are not selected. It is because these schedules may become an optimal schedule according to the arrival of new requests in the future even though they are not optimal now.

It is possible that all schedules will be removed through the above pruning conditions. For example, when the I/O subsystem is overloaded and no feasible schedule exists, all schedules may be pruned. To resolve this phenomenon, if the number of candidate schedules becomes less than threshold, G-SCAN maintains a certain number of relatively superior schedules even though they satisfy the pruning conditions. The relative superiority here is evaluated by considering both total seek time and deadline miss time of real-time requests. On the other hand, there is a possibility of incurring large overhead if too many schedules satisfy the conditions of G-SCAN. To solve this problem, we give rankings to the schedules according to the relative superiority and then cut down schedules whose ranking is beyond another threshold. Note that G-SCAN might not find an optimal schedule in the true sense of the definition. Essentially, an optimal algorithm requires the knowledge of request sequences that will arrive in the future. Our goal is to design an algorithm which can obtain a schedule close to optimal with reasonable execution overhead. The algorithm of G-SCAN is listed in Algorithms 1 and 2. ADD_REQUEST() is invoked when a new request arrives and SERVICE_REQUEST() is invoked when the disk dispatches a request in the queue for I/O service.

## 4. Performance Evaluation

### 4.1. Experimental Methodology

To assess the effectiveness of G-SCAN, we performed extensive experiments by replaying various traces collected. We compare G-SCAN with other representative on-line algorithms, namely, C-SCAN, EDF, SCAN-EDF, and Kamel's algorithm [13] in terms of the average response time, total seek distance, throughput, and deadline miss rate. We also show that the overhead of G-SCAN is feasible to be implemented. To evaluate the algorithms in various heterogeneous workload environments, we use both synthetic and real-world I/O traces. For synthetic traces, we generated four different types of workloads as shown in Table 2. Workloads 1 to 4 consist of various heterogeneous I/O workloads including real-time and best-effort applications. We modeled two different types of real-time applications based on their access patterns, namely, random and periodic. In the random type, data positions, I/O request times, and deadlines are determined randomly each time, while the periodic type has regular values. Similarly, we modeled best-effort applications as two different access patterns, namely, random and sequential.

To show the effectiveness of G-SCAN under more realistic conditions, we also performed experiments with real-world I/O traces gathered from Linux workstations (workloads 5 and 6 in Table 2). We executed the* IOZONE* program and the* mpeg2dec *multimedia player together to generate different types of I/O requests.* IOZONE* is a filesystem benchmark tool which measures the performance of a given file system. It generates various random I/O requests, and their average interarrival times in workloads 5 and 6 are 10 ms and 19 ms, respectively [25].* mpeg2dec* is a program for playing video files, which generates real-time I/O requests periodically. Average interarrival times of I/O requests generated by* mpeg2dec* in workloads 5 and 6 are about 45 ms and 90 ms, respectively. The deadline of real-time I/O requests in* mpeg2dec* is about 30 ms.

### 4.2. Effects of Grouping

**Figure 3:**
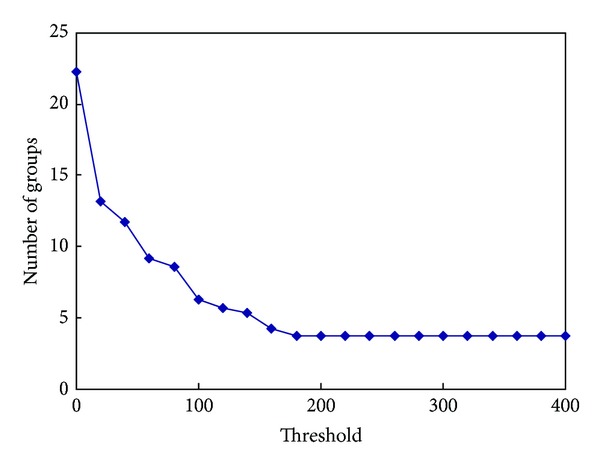
The number of groups after grouping adjacent best-effort requests. The searching space is significantly reduced by the grouping.

Before comparing the performances of G-SCAN against other algorithms, we first investigate the effect of grouping when workload 5 (real workload) is used.  Figure 3 shows the average number of groups as a function of threshold *τ*. Note that the number of groups illustrated in Figure 3 includes real-time requests as well as grouped best-effort requests. The unit of *τ* is defined as the track distance of two requests. For example, if *τ* is set to 100, best-effort requests whose track distance is smaller than 100 can belong to the same group. As can be seen from Figure 3 the searching space, namely, all possible combinations of schedules, is significantly reduced after grouping.

For example, when grouping is not used, the average number of requests in the queue is about 22 and thus the size of entire searching space is 22! which is a number larger than 10^21^. Note that the zero extreme of threshold *τ* in the graph implies that grouping is not used. However, after grouping is used, the searching space is significantly reduced. For example, when the threshold *τ* is 100 tracks, the average number of groups becomes about 6, and thus the searching space is reduced to 6! = 720. Moreover, G-SCAN does not expand this searching space completely because it also uses heuristics to reduce the searching space even more.

**Figure 4:**
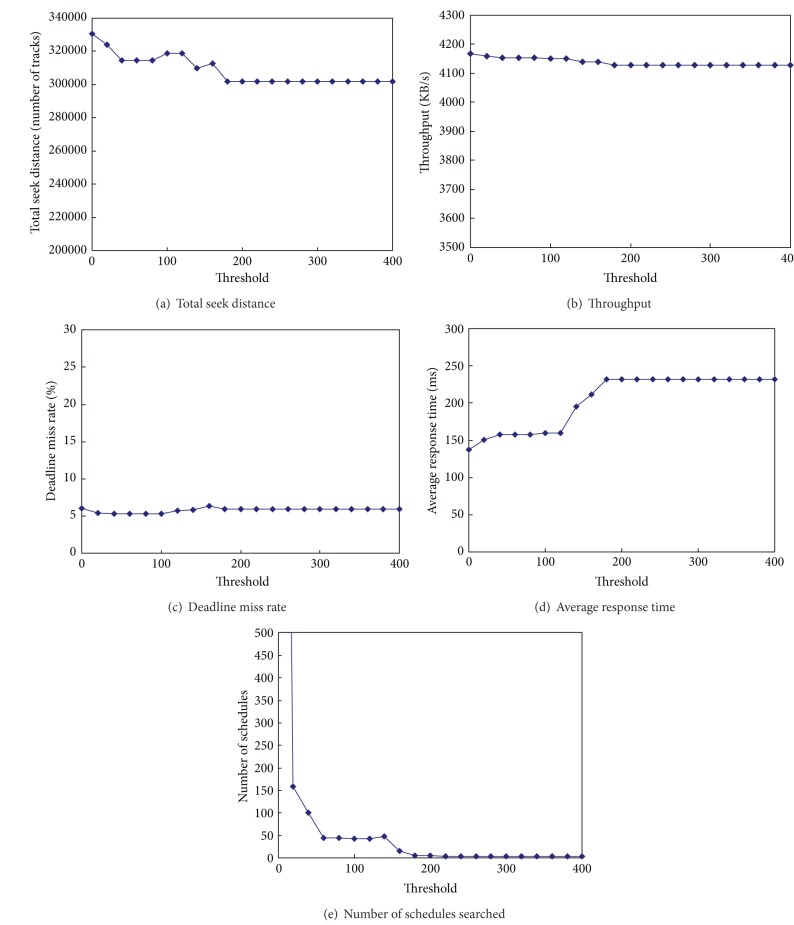
The effect of grouping in G-SCAN.

To see the effect of grouping, we investigate the performance of G-SCAN in terms of various aspects as a function of threshold *τ*. We also use workload 5 (real workload) in this experiment. As can be seen from Figures 4(a)–4(c), total seek distances, throughput, and deadline miss rate are scarcely influenced by the value of threshold *τ*. In the case of average response time, however, the performance degrades significantly when *τ* is larger than 100 as shown in Figure 4(d). We also compare the number of schedules actually expanded as a function of threshold *τ*. As can be seen from Figure 4(e), grouping significantly reduces the number of schedules to be handled. Specifically, the number of expanded schedules drops rapidly when the threshold *τ* is larger than 60. With these results, we can conclude that grouping of adjacent best-effort requests can significantly reduce the searching space without performance degradations when the threshold *τ* is set to a value around 100. In reality, finding an appropriate *τ* value for each workload environment is not an easy matter and is a topic that we are still pursuing. We use the default value of *τ* as 100 throughout this paper because it shows good performances and incurs reasonably low scheduling overhead for all workloads that we considered.

### 4.3. Performance Comparison

**Figure 5:**
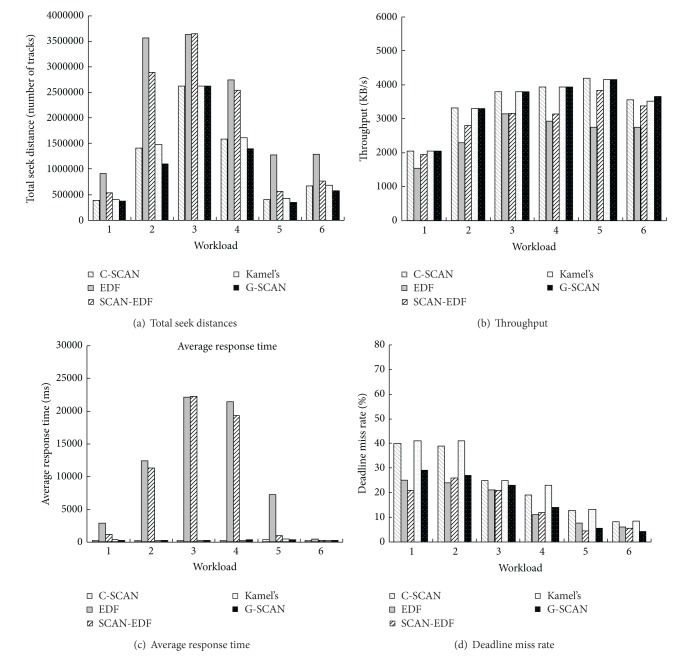
Performance comparison with various workloads.

In this subsection, we compare the performance of G-SCAN with other scheduling algorithms. We use four synthetic workloads and two real workloads listed in Table 2. Note that the performance of G-SCAN is measured when *τ* is set to 100. First, we investigate the total seek distances of the five algorithms. As shown in Figure 5(a), G-SCAN outperforms the other algorithms for all workloads that we experimented. C-SCAN and Kamel's algorithm also show competitive performances though the performance gap between G-SCAN and these two algorithms is distinguishable for workloads 2 and 4. Figures 5(b) and 5(c) show the throughput and the average response time of the algorithms, respectively. For both of the metrics, G-SCAN again performs better than C-SCAN and Kamel's algorithm. EDF and SCAN-EDF result in excessively large average response time for all cases. The reason is that EDF and SCAN-EDF greedily follow the earliest deadline irrespective of request positions.  Figure 5(d) compares the deadline miss rate of the five algorithms. As expected, deadline-based algorithms such as EDF and SCAN-EDF perform well for most cases. C-SCAN and Kamel's algorithm do not show competitive performances. G-SCAN shows reasonably good performances in terms of the deadline miss rate for all cases. Specifically, G-SCAN performs better than even EDF when real workloads (workloads 5 and 6) are used. In summary, G-SCAN satisfies the deadline constraints of real-time requests and at the same time exhibits good performances in terms of the average response time, throughput, and seek distances for both synthetic and real-world traces.

To show the upper bound of performance, we additionally measured the performance of several unrealistic algorithms that have more information to schedule, namely, OPT-D, OPT-T, and OPT-G. OPT-D is an optimal algorithm in terms of the deadline miss rate that minimizes the number of requests missing its deadline. OPT-T moves the disk head in order to minimize the total seek time irrespective of deadline misses, which performs similarly to the original SCAN algorithm. Finally, OPT-G moves the disk head to minimize the seek time and meet the deadlines of real-time requests simultaneously if a feasible schedule exists. When no feasible schedule exists, OPT-G moves the disk head to minimize the seek time. OPT-G is a complete version of G-SCAN that does not use neither grouping nor branch-and-bound scheme.

**Figure 6:**
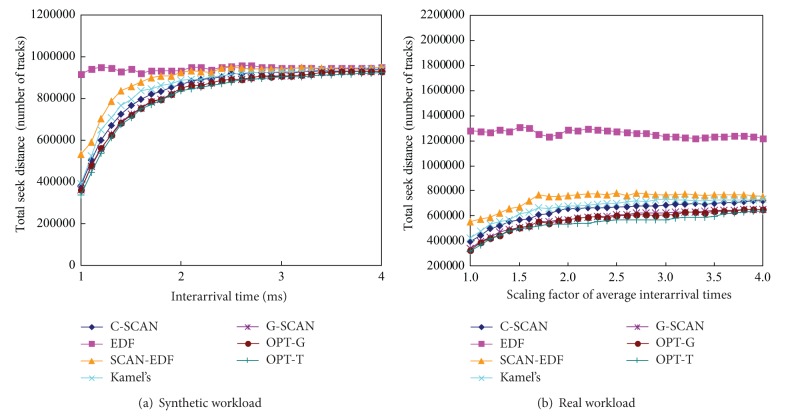
Total seek distances of the algorithms as a function of workload intensities.

**Figure 7:**
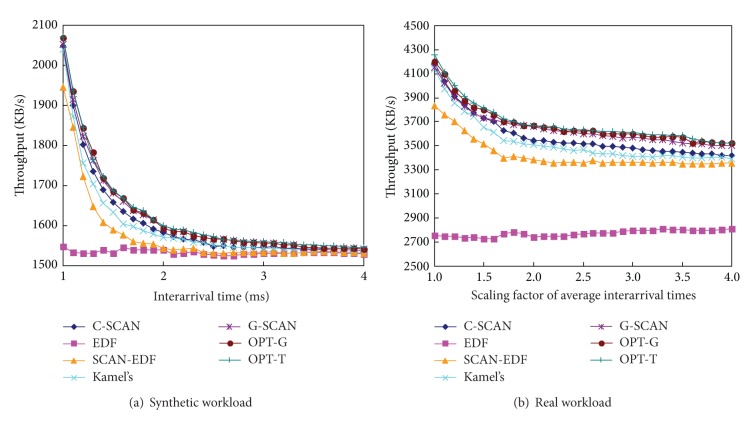
Throughput of the algorithms as a function of workload intensities.

**Figure 8:**
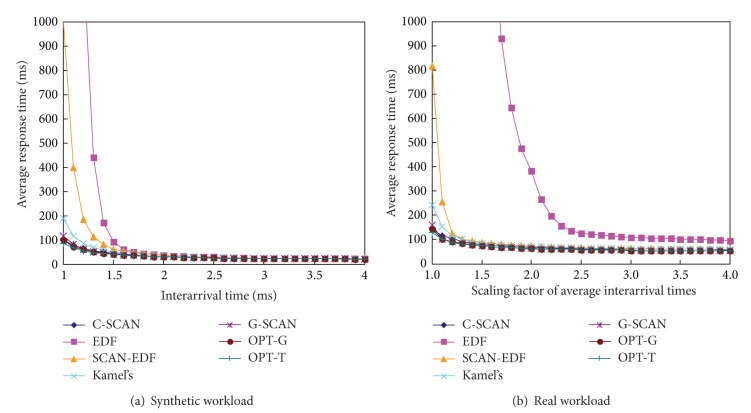
Average response time of the algorithms as a function of workload intensities.

Figures 6, 7, and 8 show the total seek distance, the throughput, and the average response time of the algorithms, respectively. The experiments were performed with workload 1 (synthetic workload) and workload 5 (real workload), respectively. We scale the original interarrival times of the workloads to explore a range of workload intensities. For example, a scaling factor of two generates a workload whose average interarrival time is twice longer than original workload. As can be seen in the figures, G-SCAN shows almost identical performances with OPT-T and OPT-G in terms of the total seek distance, the throughput, and the average response time. As expected, EDF results in extremely poor performance in terms of the three metrics because it does not consider the movement of the disk head.

**Figure 9:**
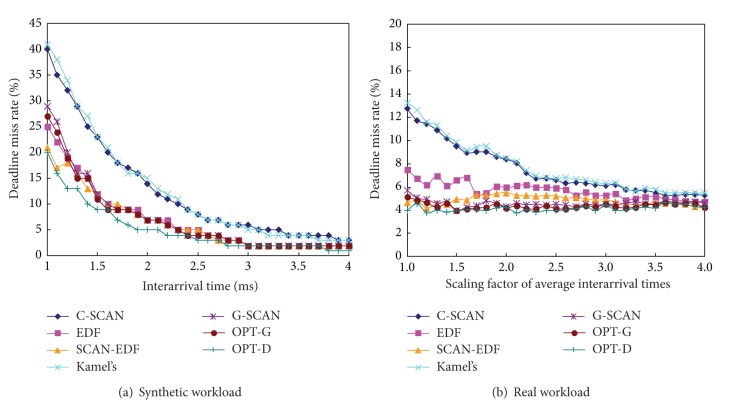
Deadline miss rate of the algorithms as a function of workload intensities.

Figure 9 compares the deadline miss rate of the algorithms. Since G-SCAN aims at reducing the seek time as well as the deadline misses, it could not exhibit better performance than OPT-D that only considers the deadline miss rate. However, G-SCAN consistently shows competitive performances in terms of the deadline miss rate. Specifically, the performance of G-SCAN is similar to that of OTP-G which pursues identical goals but does not use either grouping or pruning mechanism. Consequently, we can conclude that the grouping and the pruning mechanism of G-SCAN significantly reduce the searching space without degradation of the performance in all aspects of the total seek distance, the throughput, the average response time, and the deadline miss rate.

### 4.4. Overhead of G-SCAN

**Figure 10:**
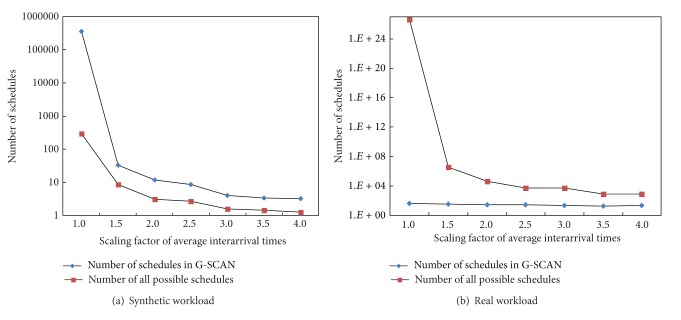
Comparison of G-SCAN and complete searching mechanism as a function of the scaling factor.

To show the overhead of G-SCAN, we measured the number of schedules expanded by G-SCAN and compared it with the number of all possible schedules.  Figure 10 shows the result for different scaling factors when workload 1 (synthetic workload) and workload 5 (real workload) are used. It is important to note that the *y*-axis in the graph is in log-scale. As shown in the figure, the number of schedules maintained by G-SCAN is reasonable for all cases. Specifically, when the scaling factor of 1.0 is used for workload 1 that refers to the original workload, the average number of schedules expanded by G-SCAN is only 298. Note that the average number of all possible schedules in this case is 7.455 × 10^15^. Similarly, the average numbers of schedules expanded by G-SCAN for real workload are smaller than 100 for all cases.

**Figure 11:**
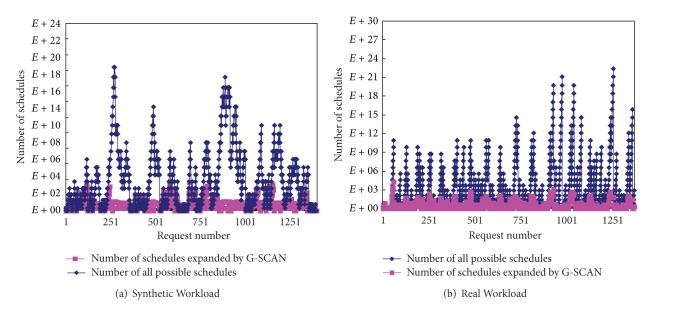
The overhead of G-SCAN as time progresses.

Figure 11 compares the schedules expanded by G-SCAN with all possible schedules when the scaling factor of 1.0 is used for workload 1 (synthetic workload) and workload 5 (real workload), respectively, as time progresses. Note that the *y*-axis is again in log-scale. As can be seen, G-SCAN explores only a small fraction of total possible schedules, and its overhead is reasonable for on-line execution.

## 5. Conclusions

In this paper, we presented a novel disk scheduling algorithm called G-SCAN that supports requests with different QoS requirements. G-SCAN reduces the huge searching space to a feasible level through grouping and branch-and-bound strategies. We have shown that G-SCAN is suitable for dealing with heterogeneous workloads since (1) it is based on the on-line request handling mechanism, (2) it meets the deadlines of real-time requests, (3) it minimizes the seek time, and (4) it has low enough overhead to be implemented. Through extensive experiments, we demonstrated that G-SCAN outperforms other scheduling algorithms in terms of the average response time, throughput, total seek distances, and deadline miss rate. We also showed that G-SCAN has reasonable overhead to be implemented for on-line execution.

## Figures and Tables

**Algorithm 1:**
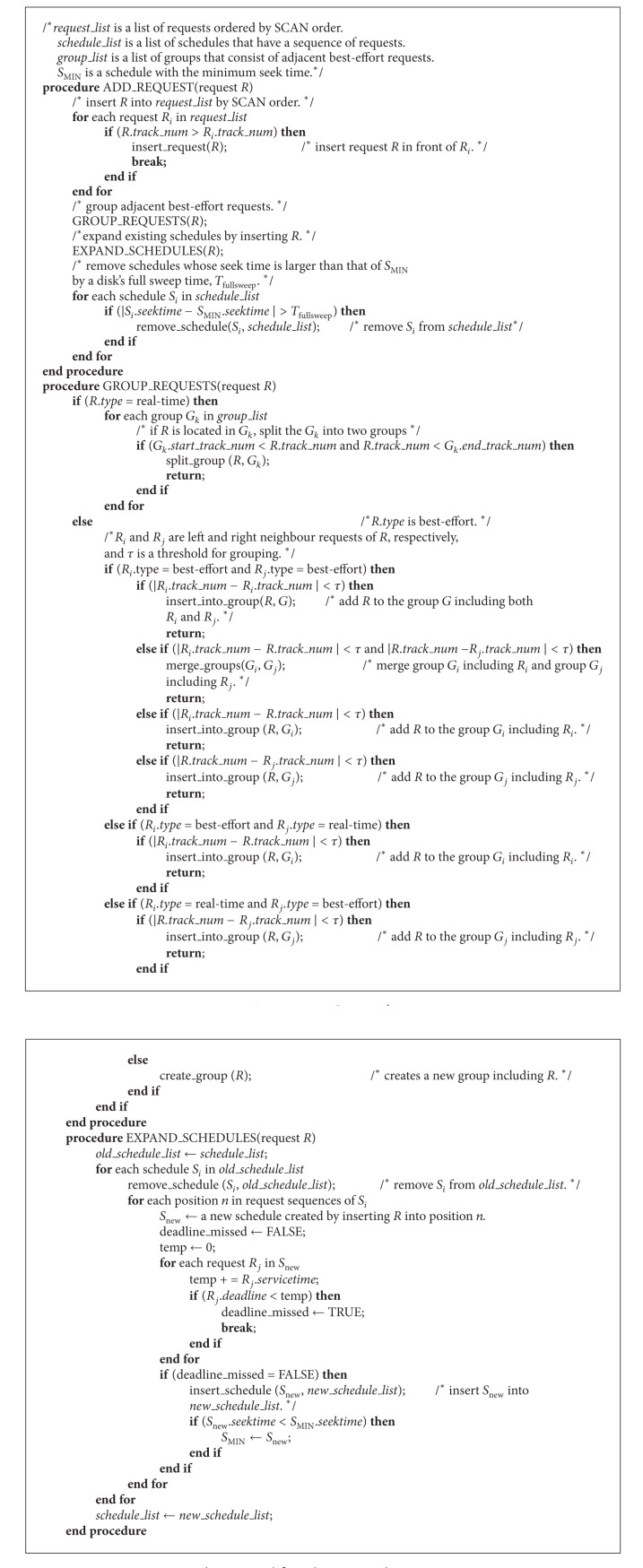
Inserting a request into queue.

**Algorithm 2:**
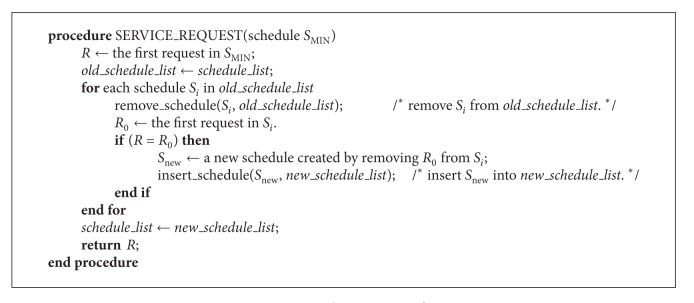
Dispatching a request from queue.

**Table 1:** A summary of disk scheduling algorithms.

Algorithm	Basic idea	Advantage	Weakness	Target applications
SSTF	Service request with shortest seek time first	Simple to implement; high throughput	High variation of response time	Best-effort applications
SATF	Service request with shortest access time (including rotational latency) first	High throughput	Require knowledge of disk structure	
SCAN	Scan in one direction and service all requests by track number order and change the direction of scan	Simple to implement; high throughput	Consider only best-effort requests	
C-SCAN	Variant of SCAN that always scans in one direction	Simple to implement; high throughput; low variation of response time	Consider only best-effort requests	

EDF	Service request with Earliest Deadline First	Simplest to implement in real-time environment	Low disk utilization	Real-time applications
SCAN-EDF	Service EDF order and use SCAN as a tie breaker	Simple to implement	Possible to degenerate into EDF	
SSEDO/ SSEDV	Consider both deadline and seek time, but put more weight on deadline	Consider both deadline and seek time	Require parameter tuning	
FD-SCAN	Move head towards the request with earliest feasible deadline; service requests on the way	Consider feasibility of real-time requests	May incur many deadline misses; high overhead	
SCAN-RT	Basically SCAN; insert new request only if it does not violate the deadlines of pending requests	Employ SCAN considering deadline	Not consider different priority level of requests	
DM-SCAN	Apply SCAN by unifying deadlines of requests within maximum scannable group	Employ SCAN considering deadline	Possible to degenerate into EDF	
Kamel's	Basically SCAN; insert new request considering deadline and priority	Consider different priority level; deadline guarantee	Immature handling of requests in next round	
MS-EDF	Find a global optimal schedule using branch-and-bound scheme	Global search of an optimal schedule; high performance	Only for real-time requests; off-line mechanism	

Rangan's	A fixed-order cyclical scheduling strategy	Employ an elaborate disk model	Not handle frame-oriented data	Multimedia streaming applications
GSS	Assign the joint deadline to each group of streams; each group is serviced in a fixed order in a round	Simple to implement; obtain high throughput by using SCAN within each round	Require group size tuning	
Preseeking sweep	Split stream data requests into multiple fragments	Obtain high throughput by employing an elaborate disk model	Require knowledge of disk structure	
Chen's	Modify round-robin scheduling to provide statistical guarantees to clients	Useful when playback guarantee is not necessary	Require complicated statistical analysis	

Cello	Two-level disk scheduling framework: a class-independent scheduler and a set of class-specific schedulers	Guarantee predefined disk bandwidth for each class	Not guarantee the jitter-free playback of multimedia	Applications with heterogeneous workloads
Reddy's	Similar to Cello; employ admission controller as well as scheduler	Consider admission controller and VBR streams	Require knowledge of disk structure	
Won's	Allocate some bandwidth to best-effort requests by extending the length of round	Consider buffer requirement for jitter-free playback of multimedia	Require knowledge of disk structure	
WRR-SCAN	Allocate disk bandwidth to prioritized task groups and service requests in the group by SCAN	Guarantee minimal disk bandwidth for aperiodic tasks	Require knowledge of disk structure	
Cascaded-SFC	Unified framework considering various scheduling parameters	Consider all scheduling parameters; applicable to various environments	Require parameter tuning	
Fahrrad	Reserve disk bandwidth based on disk time utilization	Fully reserve the disk bandwidth for different applications	Less efficient in small bursty workload with low-latency targets	
Horizon	Two-layered approach: upper level for deadline assignment and lower level for scheduling	Schedule requests based on their expected disk service time	Lack of hard real-time supports	

**Table 2:** Summary of workloads used in the experiments.

Workload	Type of application	Access pattern	Average interarrival time (ms)	Deadline (ms)	I/O size (KB)	File size (MB)
(1)	Real-time application	Random	20	30–70	64	100
	Best-effort application	Random	20	Infinite	4–128	100

(2)	Real-time application	Periodic	20	20	64	100
	Best-effort application	Random	20	Infinite	4–128	200

(3)	Real-time application	Periodic	20	20	64	100
	Best-effort application	Sequential	20	Infinite	64	200

(4)	Real-time application	Periodic	20	20	64	100
	Best-effort application	Random	45	Infinite	4–128	100
	Best-effort application	Sequential	20	Infinite	64	100

(5)	Real-time application	Periodic	45	30	4–64	324
	Best-effort application	Random	10	Infinite	4–64	128

(6)	Real-time application	Periodic	90	30	4–64	324
	Best-effort application	Random	19	Infinite	4–64	128
